# Circular RNA hsa_circ_0078607 suppresses ovarian cancer progression by regulating miR-518a-5p/Fas signaling pathway

**DOI:** 10.1186/s13048-020-00664-1

**Published:** 2020-06-05

**Authors:** Nan Zhang, Yue Jin, Qiubo Hu, Shanshan Cheng, Chao Wang, Zhiyou Yang, Yu Wang

**Affiliations:** 1 Department of Obstetrics and Gynecology, Renji Hospital, School of Medicine, Shanghai Jiaotong University, Shanghai, China; 2 Shanghai Key Laboratory of Gynecologic Oncology, Renji Hospital, School of Medicine, Shanghai Jiaotong University, Shanghai, China

**Keywords:** circ_0078607, Ovarian cancer, miR-518a-5p, Fas

## Abstract

**Background:**

Increasing researches have demonstrated the critical functions of circular RNAs (circRNAs) in the progression of malignant tumors, including ovarian cancer. In this study, we aim to investigate abnormally expression of hsa_circ_0078607 and the role of hsa_circ_0078607 during ovarian cancer pathogenesis.

**Methods:**

RT-PCR were used to detect the expression of circ_0078607 in ovarian cancer tissues. To determine the functional roles of circ_0078607 in ovarian cancer, cell proliferation and cell invasion assays were performed. Bioinformatics and luciferase reporter analysis were used to predict the target of circ_0078607.

**Results:**

In the present study, we first found that circ_0078607 was downregulated in ovarian cancer. Forced circ_0078607 expression significantly suppressed proliferation and promoted apoptosis of ovarian cancer cells. Mechanically, bioinformatics and luciferase reporter analysis identified miR-518a-5p as a direct target of circ_0078607, while Fas as a direct target of miR-518a-5p. MiR-518a-5p negatively regulated Fas in ovarian cancer cells, while overexpression of circ_0078607 could increase the expression of Fas inhibited by miR-518a-5p. Furthermore, overexpression of circ_0078607 could inhibit the proliferation and invasion of ovarian cancer cells caused by miR-518a-5p mimic.

**Conclusion:**

The results of the present study revealed that circ_0078607 suppressed ovarian cancer progression by sponging oncogenic miR-518a-5p to induce Fas expression, which may provide new therapeutic approach for ovarian cancer.

## Background

As one of the three major prevalent gynecological malignant tumors, ovarian cancer has the highest mortality rate among all gynecological cancers worldwide [1]. About 60 to 70% of patients were firstly diagnosed in the advanced stage of ovarian cancer owning to obscure typical symptoms [2]. However, the comprehensive pathogenesis of ovarian cancer remains unclear so far. Despite the advancements in surgical, chemotherapeutic and radio therapeutic treatment, the prognosis of ovarian cancer remains unsatisfactory, while its morbidity and mortality have elevated year by year [3, 4]. These challenges lead to the urgency of identifying potential biomarkers in early diagnosis.

Numerous studies have demonstrated that non-coding RNAs (ncRNAs) play important roles in tumorigenesis [5, 6]. Circular RNAs (circRNAs) are a class of ncRNAs consisting of a spectrum of conserved endogenous RNAs. They are formed by exon skipping or back-splicing events, and they can regulate gene expression via competitive binding to microRNA (miRNA) [7]. In the past few years, the biological function of circRNAs have attracted much attention, especially on the occurrence and development of cancers [8]. The most pronounced function of circRNA is miRNA sponge as a competing endogenous RNA (ceRNA) [9]. CircRNAs have been reported to bind with tumor-associated microRNAs and proteins to affect cancer progression and been used to be biomarkers for the diagnosis and prognosis of cancers [10]. Many studies have reported that circRNA has correlation with numerous cancers, including colon cancer [11], breast cancer [12], gastric cancer [13], pancreatic ductal adenocarcinoma [14] and so on, via the ceRNA network. Abnormal expression of circRNA during ovarian cancer development has also been previously reported [15]. However, the expression profile and function of circRNAs in human ovarian carcinoma remain to be investigated.

In the present study, we characterized circRNA transcripts using RNA sequencing (RNA-seq) analyses of ribosomal RNA-depleted total RNA from 9 pairs of ovarian cancer and adjacent non-cancerous tissue samples. Finally, we discovered that hsa_circ_0078607 [16] was relatively lower expressed in ovarian cancer tissues and had never been reported in tumors. Circ_0078607 is derived from exonic back-splicing of SLC22A3 gene. We subsequently expanded the sample size and detected circ_0078607 expression and found that its expression level in ovarian cancer was significantly lower than that of adjacent non-tumorous tissues. Therefore, we further explored underlying functions of circ_0078607 in ovarian cancer and speculated that circ_0078607 could serve as a sponge of miR-518a-5p to elevate Fas expression, thus inhibiting proliferation and invasion and promoting apoptosis of ovarian cancer cells.

## Material and method

### Patients and clinical tissues

This project was approved by the Ethics Committee of Renji Hospital, School of Medicine, Shanghai Jiaotong University. The ovarian cancer tissues and adjacent normal tissues samples were collected from patients diagnosed with epithelial ovarian cancer and received surgery treatment at the Department of Gynecology in Renji hospital. All the enrolled patients signed informed consents in compliance with the declaration of Helsinki. None of them had undergone chemotherapy or radiotherapy prior to surgery. All tissues were immersed in liquid nitrogen and preserved at − 80.

### RNA-seq analysis and identification and quantification of circRNAs

The libraries of RNA-seq were prepared using the NEBNext Ultra II RNA Library Prep Kit for Illumina (New England Biolabs, Beverly, MA) following its protocol. The libraries were quality controlled with a Bioanalyzer 2100 (Agilent, Santa Clara, CA) and sequenced by HiSeq 2000 (Illumina, San Diego, CA) on a 100 bp paired-end run. BOWTIE2 version 2.2.5 was used as the mapping method to the respective reference genome (GRCH37.p13 NCBI). Unmapped Reads was collected to identify the circRNA utilizing BWA mem. The strength of potential splicing sites supported by these candidate head-to-tail junction reads was then estimated using MaxEntScan33. The exact junction site was determined by selecting the donor and acceptor sites with the highest splicing strength score. Candidate circRNAs were reported if the head-to-tail junction was supported by at least two reads and the splicing score was greater than or equal to 10. To estimate the expression of circRNA, we re-aligned all the unmapped reads to the circRNA candidates by using the BWA-mem. Sequence at the 5′ end was concatenated to the 3′ end to form circular junctions. Reads that mapped to the junction were counted for each candidate. For each circRNA, we searched for the longest transcript fragment whose boundaries exactly matched both ends of this circRNA in the same strand and then defined the corresponding gene of this transcript fragment as the host gene of this circRNA.

### Cell culture

Human ovarian cancer cell lines (SKOV3 and A2780) were purchased from Cell Bank of China Academy of Sciences, Shanghai, China. All the ovarian cancer cell lines were incubated in DMEM supplemented with 10% FBS (Gibco, GranIsland, NY, USA), 100 mg/mL of penicillin and streptomycin (Invitrogen). All the cells were maintained in incubator with 5% CO_2_ and saturated humidity at 37 °C.

### RNA isolation and quantification

Total RNA from tissues and indicated treated cells were extracted with TRIzol reagent. The RNA was reverse transcribed to cDNA following the instructions of reverse transcription kit PrimeScript® RT Master Mix Perfect Real Time kit (TaKaRa, Dalian, China). Real-time quantitative reverse transcription-polymerase chain reaction (RT-PCR) was performed using SYBR Green qPCR Master Mix (Yeasen) on an ABI PRISM 7500 fast Sequence Detection System (Applied Biosystems, Foster City, CA, USA) following the protocols. Primers were designed to perform the amplification, and the sequences were as follows: GAPDH, Forward: 5′-TATGATGATATCAAGAGGGTAGT-3′ and Reverse: 5′-TGTATCCAAACTCATTGTCATAC-3′; SLC22A3, Forward: 5′-GACGTGGATGACTTGCTACG-3′ and Reverse: 5′-GGCAATTCCAGGGAGAATTA-3′; hsa_circ_0078607, Forward: 5′-CGGAATTCTGAAATATGCTATCTTACAGTTTGACCTTGTCTGTGTCAATG-3′ and Reverse: 5′-CGGGATCCTCAAGAAAAAATATATTCACCTCTGAGTAATTTGATGAGAGG-3′; miR-518a-5p, Forward: 5′-CTGCAAAGGGAAGCCCTT-3′ and Reverse: 5′-TATCCAGTGCGTGTCGTG-3′; miR-527, Forward: 5′-CAAAGGGAAGCCTTT-3′ and Reverse: 5′-TATCCAGTGCGTGTCGTG-3′; Fas, Forward: 5′-GCTGGGCATCTGGACCCTCCTACCT-3′ and Reverse: 5′-CAGTCACTTGGGCATTAACACTT-3′; GAPDH as an internal control. The comparative expression level was compared using 2^-ΔΔCt^ method.

### Cell transfection

The synthetic circ_0078607 sequence was subcloned into the pcDNA3.1 vector (Invitrogen). pcDNA3.1-circ_0078607, miR-518a-5p mimics (5′-CTGCAAAGGGAAGCCCTTTC-3′), miR-518a-5p inhibitor (5′-GAAAGGGCTTCCCTTTGCAG-3′), Fas mimic and negative controls were transfected into cultured ovarian cancer cell lines using Lipofectamine 3000 (Invitrogen, USA) according to the manufacturer’s instructions. Cells were collected 48 h after transfection and the expression levels of circ_0078607, miR-518a-5p and Fas were determined by RT-PCR.

### CCK-8 assay

Cell Counting Kit-8 (CCK-8, Beyotime, Beijing, China) was applied for testing the proliferation ability of ovarian cancer cell lines according to manufacturer’s instruction. Briefly, the treated cells were collected and inoculated in 96-well plates at a density of 2 × 10^4^cells/ml in 100 μl per well for 24,48 and 72 h. 10 μl CCK-8/well were added and incubated for 2 h at indicated time point. The absorbance value at 450 nm was measured using microplate reader (BioTek, USA). The experiment was repeated three times.

### Transwell assay

Treated ovarian cancer cell lines with 200 μL of serum-free medium were seeded in the upper chamber of each insert (8-μm pore size, Corning, USA) with Matrigel coated (BD Bioscience, San Jose, USA) for the transwell invasion assay. Lower chambers of the inserts were filled with 600 μL of medium with 10% FBS. Chambers were incubated at 37 °C in a humidified incubator containing 5% CO2 for 48 h. Then the cells invaded to the lower surface of the insert were fixed with methanol, stained by crystal violet and counted under a light microscope.

### Western blot

After indicated treatment, cells were collected. Total protein was extracted by ice-cold radioimmunoprecipitation assay (RIPA) lysis buffer (Active Motif, Carlsbad, CA) which contains a protease inhibitor cocktail (Sigma) then the protein was boiled for degeneration. After quantified using the Bradford’s method, proteins were separated in SDS-PAGE and transferred on PVDF membrane. After being electro-transferred and blocked with 3% BSA, the membranes were incubated with primary antibody: caspase3 (1:1000) (Santa Cruz), Fas (11000) (Santa Cruz). GAPDH was used as control. Enhanced chemiluminescence was used to display the bands.

### Caspase 3 activity assay

Caspase 3 activity was measured using Caspase-Glo® Assay kit (Promega, Sunnyvale, CA) according to a protocol provided by the manufacturer. Briefly, 2 × 10^4^ treated ovarian cancer cell lines were plated in each well of a 96-well plate. 48 h later 100 μL Caspase-Glo® reagent was added to each well for incubation at room temperature for 1 h. The luminescence that is proportional to caspase 3 activity was immediately measured using a microplate reader (BioTek, USA).

### Target prediction and luciferase reporter assay

The targeting relationship between circ_0078607/Fas and miR-518a-5p was respectively predicted using the Circular RNA Interactome and TargetScan website [17, 18] For dual-luciferase reporter assay, wild type (WT) and mutant (Mut) circ_0078607/FAS fragments were constructed into the vectors (Promega, Madison, WI, USA) and then co-transfected with miR-518a-5p mimic or mimics-NC into ovarian cancer cells using Lipofectamine 3000. Luciferase activity assays were performed in the light of the manufacturer’s instructions.

### Statistical analysis

All data analyses were conducted using GraphPad Prism 7 software (SanDiego, CA). Quantitative data were expressed as mean ± SD. Comparisons between two groups were analyzed using t-test and comparisons among multiple groups were analyzed by one-way ANOVA. *P* < 0.05 was considered statistically significant.

## Results

### Reduced expression of circ_0078607 in ovarian cancer tissues

**Fig. 1:**
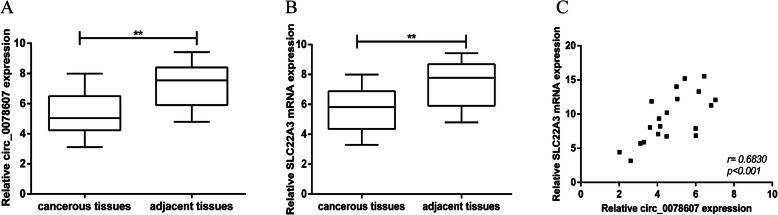
Reduced expression of circ_0078607 in ovarian cancer tissues. **a** Relative expression level of circ_0078607 in 20 ovarian cancer tissues and paired adjacent normal tissues. **b** Relative expression level of SLC22A3 mRNA in 20 ovarian cancer tissues and paired adjacent normal tissues. **c** Correlation of expression between circ_0078607 and SLC22A3 mRNA. Error bars represent mean ± standard deviation (SD). **p < 0.01

qRT-PCR analysis was performed to evaluate hsa_circ_0078607 expression in 20 pair of human ovarian cancer tissues and their corresponding adjacent non-cancerous tissue samples. The date demonstrated that circ_0078607 level was markedly reduced in ovarian cancer tissues relative to the non-cancerous counterparts (Fig. 1a). We also detected expression of linear SLC22A3 mRNA, which is the linear isomer of circ_0078607 in 20 selected ovarian cancer and adjacent non-cancerous tissues. Expression of SLC22A3 in ovarian cancer tissues was significantly lower than that in adjacent non-cancerous tissues (Fig. 1b). Additionally, positive correlation was detected between circ_0078607 and SLC22A3 mRNA (Fig. 1c).

### Circ_0078607 overexpression suppressed proliferation and promoted apoptosis of ovarian cancer cells

**Fig. 2:**
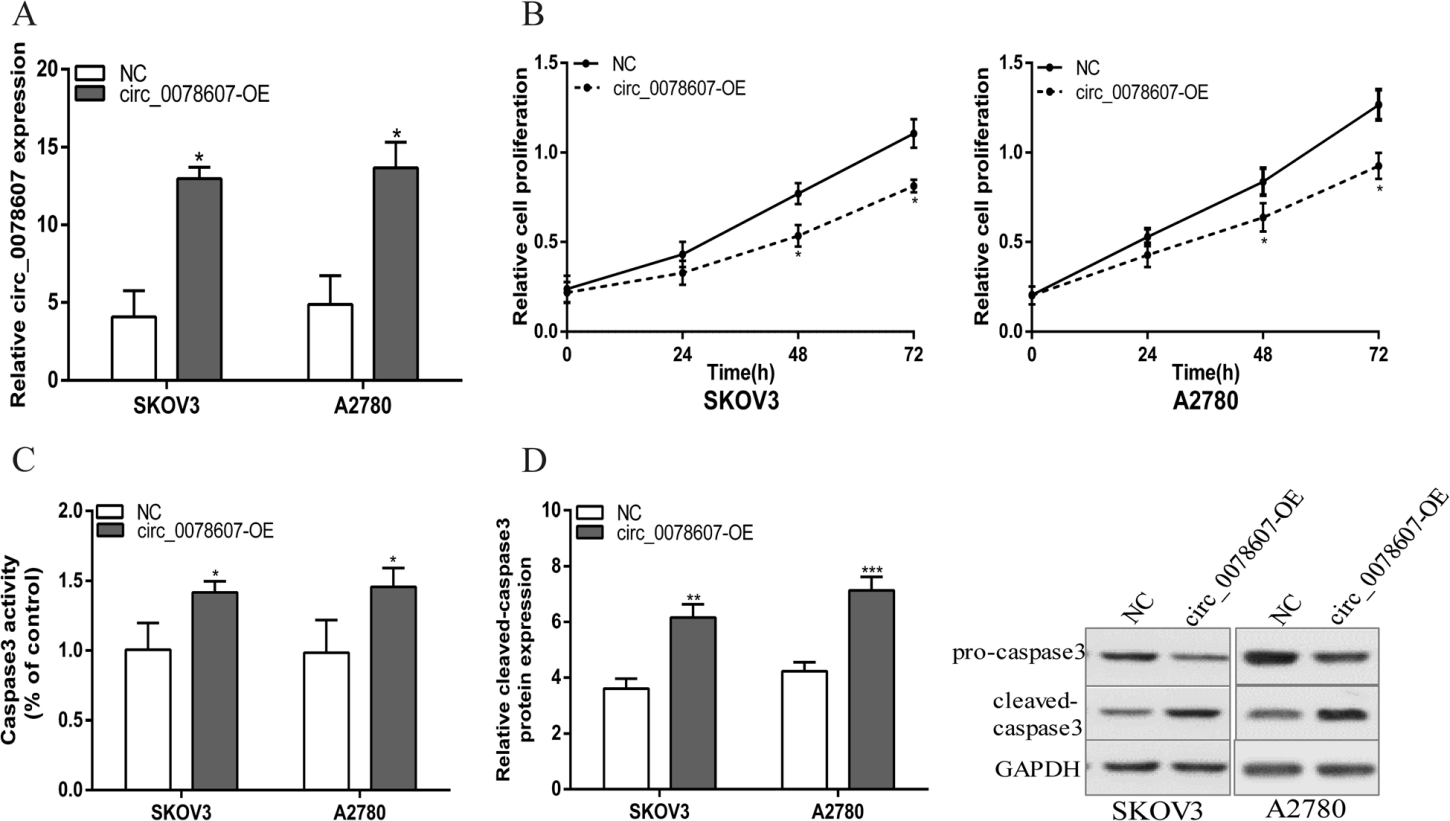
Circ_0078607 overexpression suppressed proliferation and promoted apoptosis of ovarian cancer cells. **a** Circ_0078607 expression was detected after transfection in SKOV3 and A2780 cells by qRT-PCR. **b** Overexpression of circ_0078607 suppressed SKOV3 and A2780 cells proliferation. **c** Overexpression of circ_0078607 increased caspase 3 activity in SKOV3 and A2780 cells. **d** Overexpression of circ_0078607 increased cleaved-caspase 3 protein level in SKOV3 and A2780 cells. Error bars represent the mean of three separate determinations ± standard deviation (SD). *p < 0.05. **p < 0.01. ***p < 0.001

To elucidate the effect of circ_0078607 on ovarian cancer cells behavior, SKOV3 and A2780 cells were transfected with pcDNA3.1-circ_0078607 and stable infectants were established. qRT-PCR results indicated that circ_0078607 was effectively up-regulated in ovarian cancer cells after transfected with pcDNA3.1-circ_0078607(Fig. 2a). The CCK8 experiment proved that overexpression of circ_0078607 dramatically inhibited the proliferation of ovarian cancer cells (Fig. 2b). In addition, caspase-3 activity assay and western blot experiments showed that SKOV3 and A2780 cells stably overexpressing circ_0078607 increased caspase 3 activity and cleaved-caspase 3 protein level dramatically. (Fig. 2c and d). Taken together, the above data showed that circ_0078607 suppressed proliferation and promoted apoptosis of ovarian cancer cells.

### Circ_0078607 directly bound to miR-518a-5p in ovarian cancer cells

**Fig. 3:**
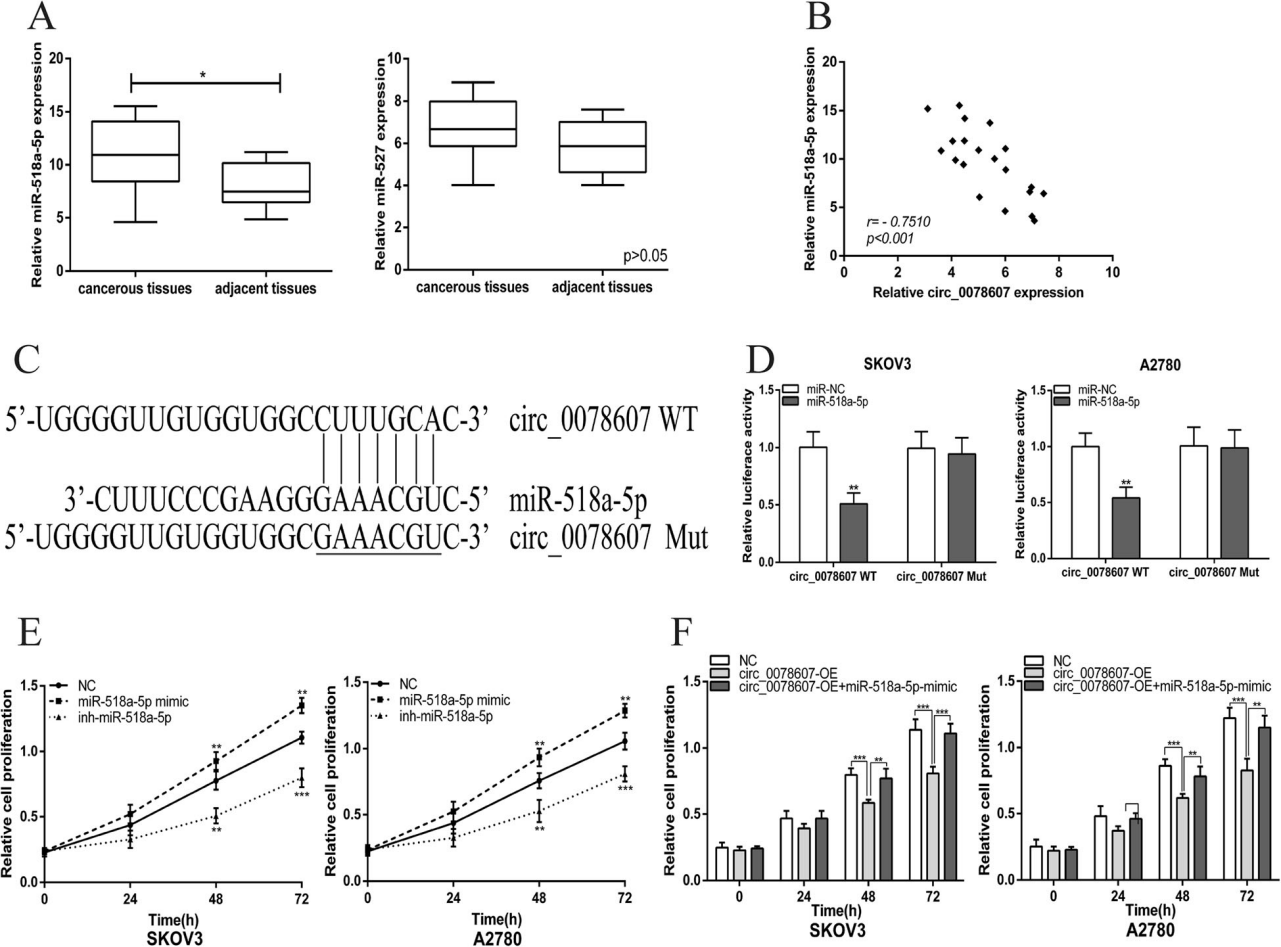
Circ_0078607 directly bound to miR-518a-5p in ovarian cancer cells. **a** Relative expression level of miR-518a-5p and miR-527 were detected by qRT-PCR in 20 ovarian cancer tissues and paired adjacent normal tissues. **b** Bivariate correlation analysis of the relationship between circ_0078607 and miR-518a-5p expression level. **c** The seed sequences of circ_0078607WT/Mut and miR-518a-5p. **d** Dual-luciferase reporter assays were performed to detect the correlation between miR-518a-5p and circ_0078607. **e** MiR-518a-5p promoted the proliferation of SKOV3 and A2780 cells. **f** MiR-518a-5p mimic functionally restored circ_0078607 overexpression-suppressed ovarian cancer cells proliferation. Error bars represent mean ± standard deviation (SD). *p < 0.05, **p < 0.01, ***p < 0.001

Next, we attempted to identify the target miRNA of circ_0078607. Bioinformatics analysis [17] suggested that circ_0078607 may bind to miR-527 and miR-518a-5p. We evaluated the expression of miR-518a-5p and miR-527 in ovarian cancer patients’ tissues and the data indicated that miR-518a-5p expression was markedly elevated in ovarian cancerous tissue samples when comparing with the normal counterparts, whereas there was no significant difference in miR-527 expression between ovarian cancerous tissue samples and the normal counterparts (Fig. 3a). Besides, a significant negative correlation was found between the expression levels of miR-518a-5p and circ_0078607 (Fig. 3b). The diagrammatic sketch of the potential binding sites obtained from bioinformatics analysis [17] for miR-518a-5p in circ_0078607 is shown in Fig. 3c. We detected interaction between circ_0078607 and miR-518a-5p using the Dual-Luciferase Reporter Assay. The results demonstrated that miR-518a-5p significantly decreased the luciferase activity of wild-type circ_0078607(WT), while miR-518a-5p had no effect on the mutant circ_0078607 (Mut) in SKOV3 and A2780 cells (Fig. 3d). We next explored the regulatory actions of miR-518a-5p in ovarian cancer cell lines. Overexpression of miR-518a-5p dramatically promoted the proliferation of ovarian cancer cells while down-expression of miR-518a-5p inhibited the proliferation of ovarian cancer cells (Fig. 3e). Besides, miR-518a-5p mimic could functionally restore circ_0078607 overexpression-suppressed ovarian cancer cells proliferation. Thus, the binding ability between circ_0078607 and miR-518a-5p was verified.

### MiR-518a-5p negatively regulated Fas in ovarian cancer cells

**Fig. 4:**
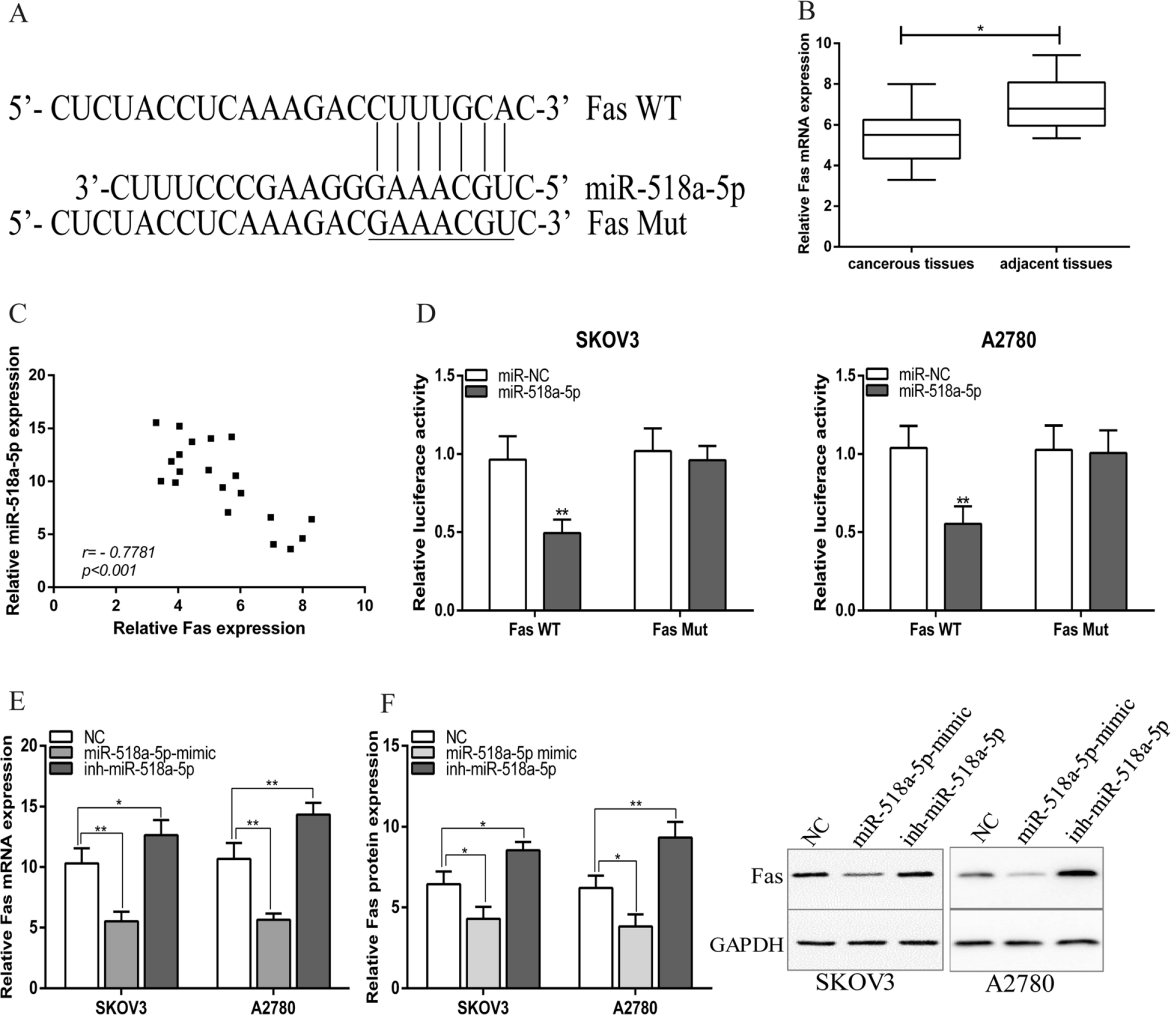
MiR-518a-5p negatively regulated Fas in ovarian cancer cells. **a** The seed sequences of Fas WT/Mut and miR-518a-5p. **b** Relative expression level of Fas were detected by qRT-PCR in ovarian cancer tissues and paired adjacent normal tissues. **c** Bivariate correlation analysis of the relationship between miR-518a-5p and Fas expression level. **d** Dual-luciferase reporter assays were performed to detect the correlation between miR-518a-5p and Fas. **e** and **f** MiR-518a-5p negatively regulates Fas expression in SKOV3 and A2780 cells. Error bars represent mean ± standard deviation (SD). *p < 0.05, **p < 0.01, ***p < 0.001

The downstream target of miR-518a-5p was predicted by TargetScan and Fas was selected for further assays. Bioinformatics analysis suggested that Fas mRNA harbored a putative miR-518a-5p binding site in its 3′-UTR (Fig. 4a). Additionally, we found that Fas mRNA expression was significantly decreased in ovarian cancer tissues compared with adjacent normal tissues (Fig. 4b) and there was a significant negative correlation between miR-518a-5p and Fas mRNA expression in ovarian cancer tissues (Fig. 4c). We used the Dual-Luciferase Reporter Assay to further explore whether miR-518a-5p directly regulates Fas expression via interaction with its 3′-UTR. We then constructed the wild-type and mutant Fas 3′-UTR reporter plasmids, SKOV3 an A2780 cells were co-transfected with miR-518a-5p or scramble, and wild-type Fas or mutated Fas respectively. The results indicated that miR-518a-5p significantly decreased the luciferase intensity in wild-type Fas, while miR-518a-5p could not change the luciferase intensity of mutant Fas group (Fig. 4d). In addition, Fas mRNA and protein expression were downregulated in miR-518a-5p-overexpressed ovarian cancer cell lines and upregulated in ovarian cancer cell lines with low expression miR-518a-5p (Fig. 4e). We concluded that miR-518a-5p negatively regulated Fas in ovarian cancer cells.

### Circ_0078607 enhanced Fas expression via sponging miR-518a-5p

**Fig. 5:**
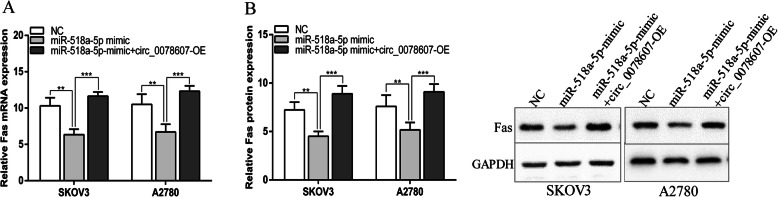
Circ_0078607 enhanced Fas expression via sponging miR-518a-5p in ovarian cancer cells. **a** and **b** The mRNA and protein level of Fas in SKOV3 and A2780 cells after transfection. Error bars represent the mean of three separate determinations ± standard deviation (SD). **p < 0.01. *** *p* < 0.001

We next explored the regulatory actions of circ_0078607 on Fas in ovarian cancer cell lines. The data showed that overexpression of circ_0078607 could enhance the expression of Fas which was inhibited by miR-518a-5p (Fig. 5a and b). We assumed that circ_0078607 was involved in enhancing the expression of miR-518a-5p downstream target Fas by functioning as a miR-518a-5p sponge.

### Circ_0078607 repressed proliferation and boosted apoptosis of ovarian cancer cells via regulating miR-518a-5p/Fas signaling

**Fig. 6:**
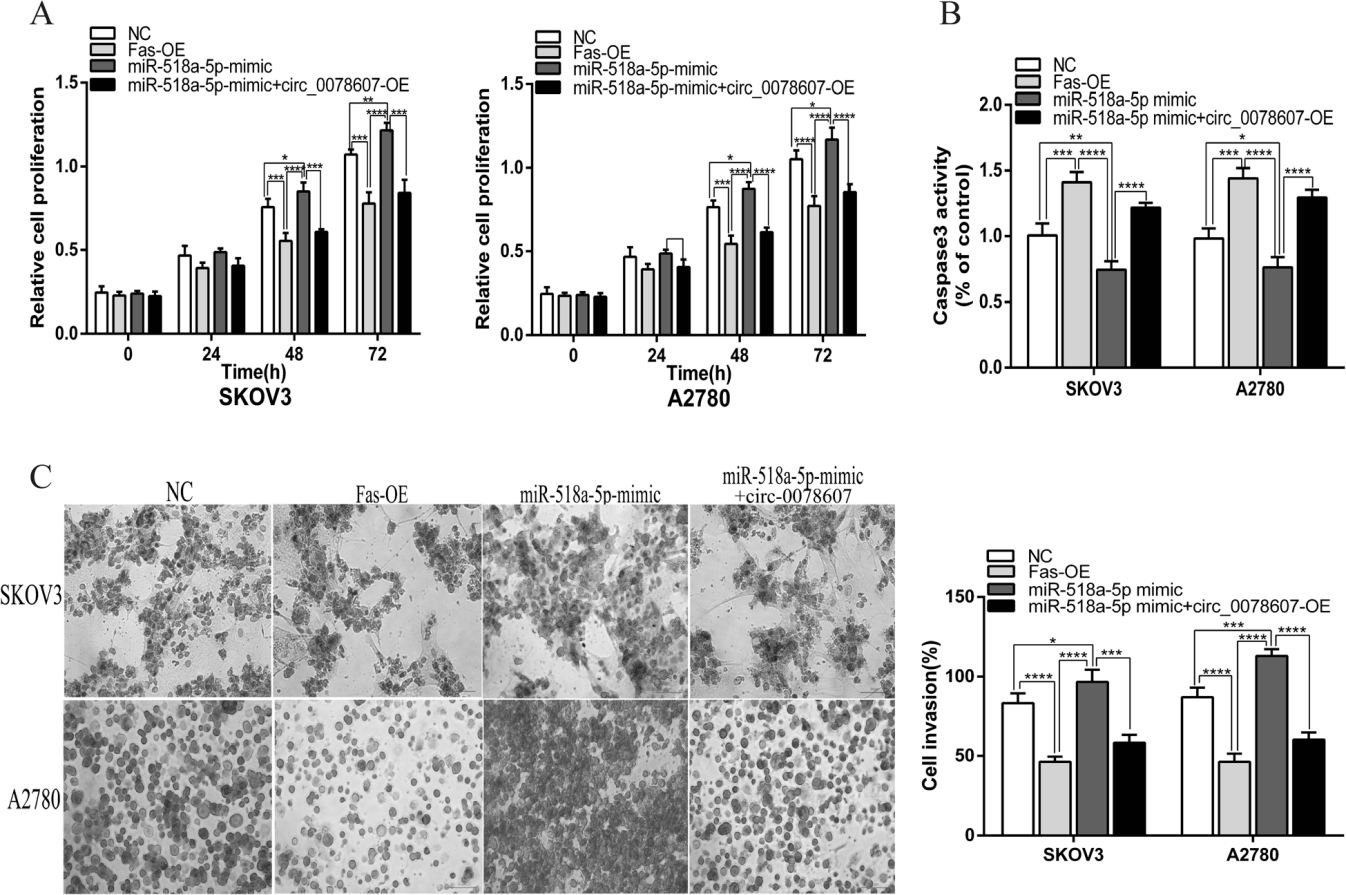
Circ_0078607 repressed proliferation and boosted apoptosis of ovarian cancer cells via regulating miR-518a-5p/Fas signaling. **a** CCK-8 assay was performed to analyze the proliferation of SKOV3 and A2780 cells under different transfections. **b** Caspase 3 activity in cells under different transfections. **c** Transwell assay was performed to detect invasion of SKOV3 and A2780 cells under different transfections. Error bars represent the mean of three separate determinations ± standard deviation (SD). *p < 0.05, **p < 0.01, *** *p* < 0.001, **** *p* < 0.0001

We demonstrated that Fas could inhibit the proliferation and invasion of ovarian cancer cells, which was the same as the effect of circ_0078607 in ovarian cancer cells, while miR-518a-5p promoted the proliferation and invasion of ovarian cancer cells. We next investigated how circ_0078607 works in miR-518a-5p mediated carcinogenesis in ovarian cancer cells. CKK-8 assay showed that circ_0078607 could inhibit the proliferation of ovarian cancer cells caused by miR-518a-5p (Fig. 6a). Consistently, circ_0078607 could inhibit the invasion of ovarian cancer cells caused by miR-518a-5p (Fig. 6c). Additionally, caspase 3 activity assay showed that reducing activity of caspase 3, which was induced by miR-518a-5p was significantly enhanced by overexpression of circ_0078607 (Fig. 6b). These data confirmed that circ_0078607 could effectively extinguish function of miR-518a-5p to suppress ovarian cancer progression. The above data implied that circ_0078607 served as a sponge of miR-518a-5p to elevate Fas expression, and to suppress cell proliferation and invasion via regulating miR-518a-5p/Fas signaling in ovarian cancer cells.

## Discussion

Ovarian cancer is one of the most common gynecologic malignancies which often presents at an advanced stage. However, the prognosis of ovarian cancer patients is still unsatisfying. Thus, a better understanding of the pathways underlying tumor progression might aid in finding out more effective treatment strategies for ovarian cancer.

The role of circRNAs in ovarian cancer has received increasing attention, multiple circRNAs, including circCSPP1 [19], circular RNA Cdr1as [20], circEXOC6B and circN4BP2L2 [21], etc., were reported to be involved in the pathogenesis of ovarian cancer and had been considered as targets for diagnosing and treating ovarian cancer. In the present study, we characterized circRNA transcripts using Ribo-free RNA-Seq from 9 pairs of ovarian cancer and adjacent non-cancerous tissue samples. Each sample was sequenced on an Illumina HiSeq yielding B60 million reads, which were mapped to the reference genome (GRCH37.p13 NCBI) by TopHat. The differentially expressed circRNAs between ovarian cancer tissue and adjacent non-cancerous tissue samples were identificated by Ribo-free RNA-Seq and annotated the known circRNA by circbase [16]. Circ_0078607 was one of the most differentially expressed circRNA from our sequencing results, which was relatively lower expressed in ovarian cancer tissues and had never been reported in tumors. The down-regulation of circ_0078607 was further identified in 20 pairs ovarian cancer tissues and adjacent non-cancerous tissue samples. This study revealed for the first time that circ_0078607 was significantly downregulated in ovarian cancer tissues. We also revealed that overexpression of circ_0078607 significantly suppressed ovarian cancer cells proliferation and invasion as well as promoted apoptosis of ovarian cancer cells.

Exploring the differentially expressed circRNAs and their functional mechanism in cancer progression has become a new research focus [22, 23]. In the present study, hsa_circ_0078607, which derived from the SLC22A3 gene, was significantly decreased in ovarian cancer tissues, and the biological functions of circ_0078607 was further investigated. The data showed that overexpression of circ_0078607 in ovarian cancer cell lines SKOV3 and A2780 remarkably suppressed cell proliferation and invasion, and stimulated cell apoptosis. Based on these results we speculated that circ_0078607 could suppress the progression of ovarian cancer but the mechanisms still need to be studied.

It’s reported that the competitive endogenous RNA (ceRNA) can affect the regulatory functions of miRNAs in gene expression and reverse the effect of miRNAs on certain pathological processes through a miRNA response element (MRE) [24, 25]. And miRNA sponge effects achieved by circRNA formation are now regarded as a general phenomenon in human malignancies [14]. Together with miRNAs and their target, the circRNA-miRNA-mRNA axis may function as an extensive regulatory network in gene expression, and their deregulation may cause disease progression including cancer development. To better understand the regulatory mechanism of circ_0078607 in ovarian cancer, we analyzed the miRNAs known to be bound by circ_0078607, bioinformatics analysis identified that miR-518a-5p was a hsa_circ_0078607 associated miRNA and the sponge adsorption effect of circ_0078607 on miR-518a-5p was further verified by dual-luciferase reporter gene assay. Previously, miR-518a-5p had been identified as a tumor regulator in many malignancies, including colorectal cancer [26], gastrointestinal stromal tumors [27] and germ cell tumors [28]. We found that miR-518a-5p was up-regulated in ovarian cancer tissues and correlated negatively with circ_0078607 in ovarian cancer tissues. Besides, miR-518a-5p could promot proliferation of ovarian cancer cells. Further functional studies showed that miR-518a-5p mimic could functionally restore circ_0078607 overexpression-suppressed ovarian cancer cells proliferation. These results suggested that overexpression of circ_0078607 inhibited ovarian cancer progression though acting as miR-518a-5p sponge.

Fas is a membrane protein that belongs to the tumor necrosis factor receptor superfamily. The interaction between Fas and its receptor Fas ligand (FasL) induces the death signal cascade, eventually leads to cell apoptosis [29]. Existing data have shown that the downregulation of Fas expression could be detected in many types of human tumors [30, 31], and the dysregulation of Fas has been demonstrated to be related to the risk of developing ovarian cancer as well as to the survival of patients treated with platinum-based chemotherapy [32]. In our study, Fas was forecasted to be the direct target of miR-518a-5p by bioinformatics analysis [18]. RT-PCR demonstrated that Fas expression was significantly decreased in ovarian cancer tissues and was negatively correlated with miR-518a-5p expression. In addition, this study proved that Fas could inhibit the proliferation and invasion of ovarian cancer cells and increased the apoptosis of ovarian cancer cells. This phenomenon was the same as the effect of circ_0078607 in ovarian cancer cells. We further demonstrated that miR-518a-5p mimic downregulated Fas mRNA and protein expression while miR-518a-5p inhibitor upregulated Fas mRNA and protein expression in ovarian cancer cells. In addition, overexpression of circ_0078607 could significantly up-regulate the expression of Fas which was inhibited by miR-518a-5p. So, we speculated that circ_0078607 acted as a sponge of miR-518a-5p to elevate Fas expression to inhibit proliferation and invasion and promote apoptosis of ovarian cancer cells. Thus, circ_0078607/miR-518a-5p/Fas network may facilitate a novel aspect of the treatment of patients with ovarian cancer. However, further investigation should be made on nude mice to verify our conclusion in vivo.

## Conclusions

This study first identified that circ_0078607, which was down expression in ovarian cancer, competitively regulated the expression of Fas by capillary adsorption of miR-518a-5p, also inhibited ovarian cancer proliferation by increasing the expression of Fas. Our discovery enriches the research of the molecular biological mechanism of circRNA involved in the development of ovarian cancer, and provides novel insights for new diagnostic and therapeutic strategies for clinical prevention and treatment of ovarian cancer.

## Data Availability

All data generated or analyzed during this study are included in this published article or are available from the corresponding author on reasonable request.
